# Advancing heart health in North Carolina primary care: the Heart Health NOW study protocol

**DOI:** 10.1186/s13012-015-0348-4

**Published:** 2015-11-14

**Authors:** Bryan J. Weiner, Michael P. Pignone, C. Annette DuBard, Ann Lefebvre, Janet L. Suttie, Janet K. Freburger, Samuel Cykert

**Affiliations:** Department of Health Policy and Management, CB 7411, University of North Carolina at Chapel Hill, Chapel Hill, NC 27599 USA; Division of Internal Medicine, CB 7110, University of North Carolina at Chapel Hill, Chapel Hill, NC 27599 USA; Community Care of North Carolina, 2300 Rexwoods Drive, Suite 100, Raleigh, NC 27607 USA; North Carolina Area Health Education Program, CB 7165, University of North Carolina at Chapel Hill, Chapel Hill, NC 27599 USA; Cecil G. Sheps Center for Health Services Research, CB 7590, University of North Carolina at Chapel Hill, Chapel Hill, NC 27599 USA

**Keywords:** Practice facilitation, Academic detailing, Regional learning collaboratives, Primary care practice, Cardiovascular disease prevention, Practice capacity, Quality improvement

## Abstract

**Background:**

The objective of Heart Health NOW (HHN) is to determine if primary care practice support—a comprehensive evidence-based quality improvement strategy involving practice facilitation, academic detailing, technology support, and regional learning collaboratives—accelerates widespread dissemination and implementation of evidence-based guidelines for cardiovascular disease (CVD) prevention in small- to medium-sized primary care practices and, additionally, increases practices’ capacity to incorporate other evidence-based clinical guidelines in the future.

**Methods/design:**

HHN is a stepped wedge, stratified, cluster randomized trial to evaluate the effect of primary care practice support on evidence-based CVD prevention, organizational change process measures, and patient outcomes. Each practice will start the trial as a control, receive the intervention at a randomized time point, and then enter a maintenance period 12 months after the start of the intervention. The intervention will be randomized to practices in one of four strata defined by region of the state (east or west) and degree of practice readiness for change. Seventy-five practices in each region with a high degree of readiness will be randomized 1:1:1 in blocks of 3 sometime prior to month 8 to receive the intervention at month 9, 11, or 12. An additional 75 practices within each region that have a low degree of readiness or are recruited later will be randomized 1:1 in blocks of 2 prior to month 13 to receive the intervention at month 14 or 16. The sites will be ordered within each strata based on time of enrollment with the blocking based on this ordering. Evaluation will examine the effect of primary care practice support on (1) practice-level delivery of evidence-based CVD prevention, (2) patient-level health outcomes, (3) practice-level implementation of clinical and organizational changes that support delivery of evidence-based CVD prevention, and (4) practice-level capacity to implement future evidence-based clinical guidelines.

**Discussion:**

Results will indicate whether primary care practice support is an effective strategy for widespread dissemination and implementation of evidence-based clinical guidelines in primary care practices. Discernible reductions in cardiovascular risk in 300 practices covering over an estimated 900,000 adult patients would likely lead to prevention of thousands of cardiovascular events within 10 years.

**Trial registration:**

ClinicalTrials.gov NCT02585557

**Electronic supplementary material:**

The online version of this article (doi:10.1186/s13012-015-0348-4) contains supplementary material, which is available to authorized users.

## Background

Cardiovascular disease (CVD) is the leading cause of death in the USA [1]. CVD imposes a staggering burden whether measured in life-years lost, diminished quality of life, racial and ethnic health disparities, or direct and indirect healthcare costs. Yet, the Centers for Disease Control and Prevention (CDC) estimates that more than 200,000 deaths from heart disease and stroke could be prevented each year through a concerted, national effort to control CVD risk factors such as blood pressure, cholesterol, and tobacco use.

In September 2011, the US Department of Health and Human Services launched the “Million Hearts” initiative to prevent 1 million heart attacks and strokes by 2017 [2]. In the clinical realm, Million Hearts focuses on the management of the “ABCS”—aspirin use in high-risk individuals, blood pressure control, cholesterol management, and smoking cessation [2]. To support Million Hearts, the US Agency for Healthcare Research and Quality (AHRQ) launched an initiative in 2015 to assist primary care practices in implementing clinical and organizational changes to improve the management of ABCS.

### Objectives and aims

Heart Health NOW (HHN), an AHRQ-funded demonstration and evaluation, investigates whether a comprehensive evidence-based quality improvement strategy (which we call primary care practice support) accelerates the implementation of evidence-based guidelines for CVD prevention in primary care practices on a large scale. The specific aims are to evaluate the effect of primary care practice support in small- to medium-sized practices on (1) practice-level delivery of evidence-based CVD prevention, (2) patient-level health outcomes, (3) practice-level implementation of clinical and organizational changes that support delivery of evidence-based CVD prevention, and (4) practice-level capacity to implement future evidence-based clinical guidelines. HHN is a cooperative effort of the University of North Carolina at Chapel Hill (UNC-CH), the North Carolina Area Health Education Center (NC AHEC), and Community Care of North Carolina (CCNC), the primary care case management provider for NC Medicaid.

## Methods/Design

### Study setting

North Carolina is the tenth largest state in the USA with a population approaching 10 million and a racial and ethnic composition of 72 % white, 22 % black, and 9 % Hispanic [3]. The burden of cardiovascular disease in North Carolina remains large. The cardiovascular death rate was 263 per 100,000 as measured in 2008–2010, accounting for almost one-third of deaths in the state. These data rank North Carolina 32nd among the 50 states for cardiovascular outcomes [4].

### Research design

HHN is a stepped wedge, stratified, cluster randomized trial. Each practice will start the trial as a control, receive the primary care practice support intervention at a randomized time point, and then enter a maintenance period 12 months after the start of the intervention. The intervention will be randomized to practices in one of four strata defined by region of the state (east or west) and degree of practice readiness for change (described below). Specifically, 75 practices in each region with a high degree of readiness will be randomized 1:1:1 in blocks of 3 prior to month 8 to receive the intervention at month 9, 11, or 12. An additional 75 practices within each region that have a low degree of readiness or are recruited later will be randomized 1:1 in blocks of 2 prior to month 13 to receive the intervention at month 14 or 16. The sites will be ordered within each stratum based on time of enrollment with the blocking based on this ordering. The study’s statistician will generate the stratified permuted-block random allocation lists using a computer program. As practices are enrolled, the project management system will use the randomization schedule to randomize practices and assign their intervention start date. A practice is considered randomized once an intervention start date has been assigned. Neither study personnel nor participating practices will be blind to randomized start dates.

### Practice recruitment

We will recruit 300 small- to medium-sized primary care practices. To be eligible, practices must have ten or fewer providers in a single location and have an electronic health record (EHR). Enrolling practices agree to the following: (1) form a quality improvement team; (2) meet with the practice facilitator up to 8 h a month; (3) send representatives to regional learning collaboratives three times yearly; (4) meet with experts to discuss CVD prevention, CVD risk assessment, and system change strategies; (5) permit the CCNC Informatics Center to process EHR data and provide the practice with cardiovascular care registries and dashboards for clinical decision support and population health management; (6) review data on ABCS measures at least monthly; and (7) use those data to plan, conduct, and assess improvement activities. Enrolling practices complete an information technology and quality improvement readiness assessment, which is used for earlier randomization of high-readiness practices and assignment of practices to more or less intensive practice facilitation.

### Primary care practice support intervention

Practice facilitators will work for 12 months with practices’ quality improvement teams to implement four “key drivers” for supporting delivery of evidence-based CVD prevention: enhanced clinical information systems with dashboards on critical measures and drill downs to patient-level care; adoption and use of practice-wide evidence-based protocols; effective use, documentation, and tracking of patient self-management support goals; and optimization of the practice care team. Practices will receive up to 8 h a month of practice facilitation depending on their level of readiness at enrollment. In addition, physician faculty will engage in academic detailing through webinars and practice visits. Detailing will focus on CVD prevention and treatment, CVD risk assessment, and new recommendations for cholesterol and hypertension management. Finally, providers and staff will participate in regional learning collaborative meetings where they can share experiences implementing evidence into practice.

### Evaluation framework

**Fig. 1:**
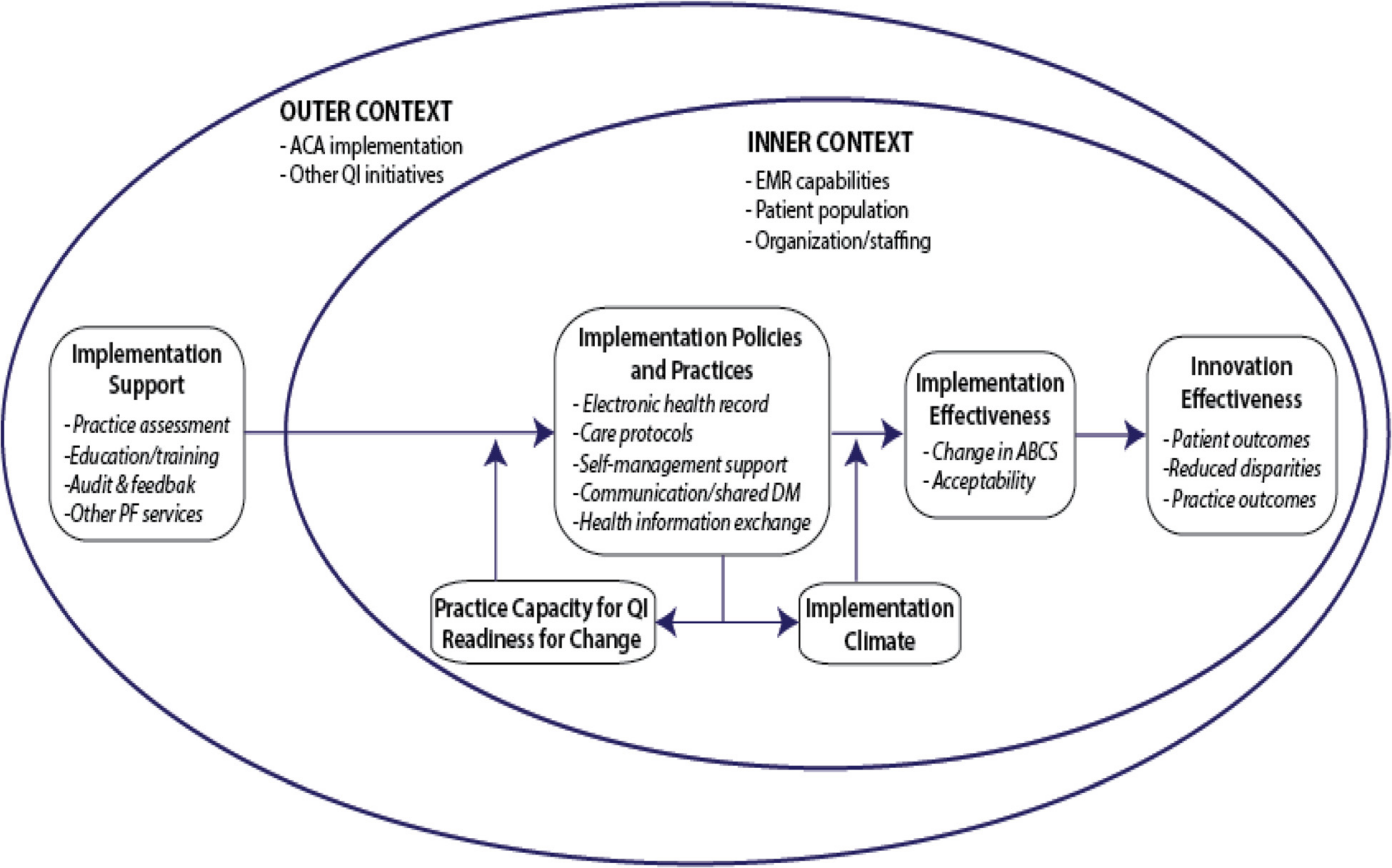
Conceptual framework guiding the evaluation of Heart Health Now

Our evaluation draws on an organizational model of innovation implementation [5–9]. The innovation here is evidence-based CVD prevention (including clinical decision support and population health management tools, global risk-based treatment of lipids, new treatment options, and evidence-based smoking cessation). Briefly, effective implementation is a function of the implementation support the practice receives and the policies and practices it employs to support innovation use (see Fig. 1). Practice capacity for QI, readiness for change, and implementation climate moderate the relationship between support, policies and procedures, and effectiveness. Inner and outer contextual factors influencing organizational priorities, capabilities, and opportunities also moderate the relationship. Realization of patient- and practice-level benefits depends on effective implementation of evidence-based CVD prevention.

### Data collection

Table 1 outlines data collection, measures, sources, and timing for each construct.

**Table 1:** Theoretical constructs, measures, data sources, and data collection timing

Construct	Measures	Source	Timing
Implementation support	Frequency, duration, and mode of PF contacts	PF contact logs	I
	Frequency, duration, and mode of academic detailing contact	Contact, webinar logs	I
	Attendance at regional collaborative meetings	Attendance logs	I
Practice capacity for QI	Change Process Capacity Questionnaire	KI survey	B, E, F
	Adaptive Reserve Questionnaire	Provider/staff survey	B, E, F
Organizational readiness	Organizational Readiness for Change Questionnaire (ORIC)	Provider/staff survey	B
Implementation Policies and Practices (IPPs)	Key Driver Implementation Scales	PF Ratings	I
	Implementation barriers, facilitators, and IPPs	KI interview	B, E
Implementation climate	Implementation climate questionnaire	Provider/staff survey	E, F
Implementation effectiveness	ABCS measures/clinical measures	HIE	B, I, E, F
	Acceptability of implementation support	KI interview	E
Innovation effectiveness	Patient outcomes (communication, shared decision-making)	Patient survey	B, E
	Patient outcomes (healthcare utilization and mortality)	Claims data	B, E, F
	Practice outcomes (e.g., financial benefits)	KI Interview	E, F
Inner context	Practice characteristics, patient population, EMR capabilities	KI survey, PF contact logs	B, E,F
Outer context	External policies and incentives, market conditions	KI survey, KI interview, PF contact logs	B, E, F

Because readiness is conceived as an organization-level construct, we will test whether sufficient inter-rater reliability and inter-rater agreement exist to aggregate individual responses to the practice level [22–26]. If tests do not justify aggregation, we will use a measure of intra-practice variability in readiness rather than a practice-level mean in our analysis [15, 16].

Source: *PF* practice facilitator, *KI* key informant; timing: *B* baseline, *I* intervention, *E* end of intervention, *F* 6 and 12 months post-intervention

#### Practice facilitator contact logs

Practice facilitators (PFs) will record monthly the frequency, duration, and mode of practice contact. We will use these data to measure the intensity of implementation support provided to practices.

#### Academic detailing logs

Physician faculty will record visits, calls, and other contacts with practices. Webinar software will track logins and pages viewed by practice staff.

#### Regional collaborative attendance records

Project staff will keep records of the number and types of staff from each practice that attend regional collaborative meetings.

#### Key informant surveys

We will survey key informants (KIs) to assess practice capacity for QI, inner context, and outer context. The surveys will be administered at baseline, end-of-intervention, 6 months post-intervention, and 12 months post-intervention for practices enrolled earlier in the study. The KI will be the practice’s provider champion (*N* = 300).

#### Provider/staff survey

We will survey providers and staff to assess practice capacity for QI at baseline, end-of-intervention, 6 months post-intervention, and 12 months post-intervention for practices enrolled earlier; organizational readiness to implement change at baseline; and implementation climate at end-of-intervention and 6 months post-intervention. The sample will consist of 3–5 providers and staff members depending on practice size (*N* = 900–1500).

#### PF ratings

PFs will assess on a monthly basis practices’ progress in implementing clinical practice and organizational changes to support improvement in CVD prevention (implementation policies and practices). Ratings will focus on the extent to which practices have implemented four key drivers of improved CVD prevention and also level of leadership and team engagement.

#### Interviews

We will conduct semi-structured KI interviews (*N* = 300) at end-of-intervention and 6 months post-intervention to obtain supplemental data about organizational changes for evidence-based CVD prevention (implementation policies and practices) and influential inner and outer context events. The KI interview at end-of-intervention will also ask about acceptability of the implementation support received (implementation effectiveness). The KI interview 6 months post-intervention will ask about practice-level outcomes (innovation effectiveness).

#### EHR/informatics

We will obtain performance data on the ABCS measures (implementation effectiveness) and patients’ intermediate clinical outcomes (innovation effectiveness) from the EHR. The ABCS measures and clinical outcomes will be collected at baseline, during the intervention (quarterly), at the end of the intervention, and post-intervention (6 and 12 months follow-up). The EHR will also supply race/demographic data. EHR data will be routed to the CCNC Informatics Center, where the data will be extracted, normalized, and placed in a standard report used for the evaluation and sent back to the practices.

#### Patient surveys

We will survey 200 consecutive adult patients in each practice at baseline and at the end of the intervention to obtain patient experience data on communication and shared decision-making (innovation effectiveness). Data will be collected during an office visit using paper-based surveys. Eligible patients must have one or more of the following comorbidities: hypertension/high blood pressure, myocardial infarction/heart attack, angina/coronary heart disease, stroke, smoking, diabetes, and high cholesterol.

#### Claims data

We will use claims data at baseline, end-of-intervention, and 6 months post-intervention to assess hospitalizations for cardiovascular-related conditions, in-hospital deaths, and mortality for patients seen at participating practices (innovation effectiveness). We will have access to claims data on 85 % of the insured population in NC, specifically those covered by Blue Cross Blue Shield of North Carolina, the State Employee Health Plan, Medicare, and Medicaid. Outcomes will be aggregated to the practice level. For the baseline period, we will examine claims data for the year prior to the intervention. Following the intervention, we will examine available claims during and after the intervention for each practice.

## Measures

*Implementation support*. We will measure primary care practice support intensity as the percentage of contacts with a practice that are in-person. We will also report the actual number of in-person contacts and number of total contacts to account for variability in contacts across practices. We will also track frequency, mode, and duration of contact with academic detailers, as well as number and type of staff per practice attending collaborative meetings.

*Practice capacity for QI* encompasses two constructs. The first construct, change process capability, refers to a “state of preparedness for change that is influenced by the organization’s previous history of change, its plans for continuous organizational refinement, and its ability through its social and technical systems to initiate and sustain that change” ([10], p. 193). We will measure this construct using the Change Process Capacity Questionnaire (CPCQ) [10]. The second construct, adaptive reserve, refers to “the practice relationship infrastructure; alignment of management functions in which clinical care, practice operations, and financial functions and reflect a consistent vision; facilitative leadership; teamwork; sense-making; a positive work environment; and a culture of learning”([11], p. S37). We will measure this construct using the Practice Adaptive Reserve scale [11, 12].

*Organizational readiness for change* refers to the extent to which organizational members are psychologically and behaviorally prepared to implement change [6, 7, 13]. We will measure readiness using the Organizational Readiness for Implementing Change scale [13].

*Implementation policies and practices* are the strategies that an organization puts into place to support innovation use [9, 14]. We will measure implementation policies and practices using the Key Drivers of Implementation Scales (KDIS) [15]. PFs will rate the extent of practice implementation of the four key drivers using a 5-point, behaviorally-anchored scale. Table 2 offers an example of the KDIS for planned care.

**Table 2:** Key Drivers of Implementation Scale (KDIS) for standardized care processes

Level	Description
0 - No activity	No activity on standardization.
1 - Protocols identified	Practice identifies sample protocols and customizes them for their practice.
2 - Staff enabled	Practice enables staff to perform roles (via standing orders, etc.) needed to implement protocols.
3 - Testing workflow	Practice is develops/tests workflows to support protocols.
4 - Implementation	Practice implements and follows at least one protocol.
5 - Full implementation	Practice monitors the system to ensure that protocols are used consistently.

*Implementation climate* refers to organizational members’ “shared summary perception of the extent to which their use of a specific innovation is rewarded, supported, and expected within their organization” ([16], p. 1060). We will measure implementation climate using a 6-item scale [17].

*Implementation effectiveness* refers to the consistency and quality of aggregate or pooled innovation use (e.g., evidence-based CVD prevention) [14]. We will assess implementation effectiveness using nine ABCS measures (see Table 3).

**Table 3:** Process of care measures for ABCS

Ischemic vascular disease (IVD): use of aspirin or another antithrombotic	Percentage of patients aged 18 years and older with ischemic vascular disease (IVD) with documented use of aspirin or other antithrombotic.
Aspirin for the primary prevention of cardiovascular disease	Percentage of patients between the ages of 50 and 75 with an ASCVD risk score of ≥10 % who do not currently have ischemic vascular disease with documented aspirin use.
Blood pressure management: controlling high blood pressure	Percentage of patients aged 18 through 85 years of age with a diagnosis of hypertension (HTN) whose blood pressure (BP) was adequately controlled (<140/90) during the measurement year.
Blood pressure management: controlling high blood pressure	Percentage of patients aged 18 through 85 years of age with a diagnosis of HTN whose blood pressure was adequately controlled ( age 18–59 and/or people with diabetes or chronic kidney disease <140/90; age 60–85 < 150/90 ) during measurement year.
Statin therapy for prevention and treatment of cardiovascular disease	Percentage of the following patients—all considered at high risk of cardiovascular events—who were prescribed or were on statin therapy: adults ages ≥21 years who were previously diagnosed with or currently have an active diagnosis of clinical atherosclerotic cardiovascular disease (ASCVD); or adults ages ≥21 years with a fasting or direct low-density lipoprotein cholesterol (LDL-C) level ≥190 mg/dL; or adults ages 40–75 years with a diagnosis of diabetes with a fasting or direct LDL-C level of 70–189 mg/dL.
Risk-based statin therapy	Percentage of patients aged 40 through 79 years of age whose Calculated Risk Score is 10 % or greater and are on a statin.
Assessment of cardiovascular risk	Percentage of patients ages 40–79, with no evidence of prior cardiovascular event, who have documentation of required elements for cardiovascular risk assessment.
Tobacco use screening	Percentage of patients aged 18 years or older who were seen for at least 2 office visits within 12 months and who were queried about tobacco use one or more times within 24 months PQRS 226 Part A (modified).
Smoking cessation support	Percentage of patients aged 18 years and older identified as tobacco users within the past 24 months and seen for at least 2 office visits within 12 months who received cessation intervention within 24 months PQRS 226 Part B (modified).

The ischemic vascular disease use of aspirin, blood pressure management 1, tobacco use screening, and smoking cessation support measures are PQRS measures with defined measure specifications [27]. The aspirin for primary prevention, blood pressure management 2, statin for primary prevention, risk-based statin therapy, and cardiovascular risk assessment measures reflect newer evidence-based guidelines

We will measure acceptability of primary care practice support qualitatively by asking about KIs’ satisfaction with the frequency, duration, and quality of implementation support.

*Innovation effectiveness* refers to the benefits that result from innovation use (e.g., patient and practice outcomes) [14]. To capture patients’ assessment of communication, we will use selected items from the CAHPS clinician and group survey (see Table 4) [18].

**Table 4:** Communication questions

In the last 12 months, how often did your provider		Response options
Q14	explain things in a way that was easy to understand?	•Never
Q15	listen carefully to you?	•Sometimes
Q17	give you easy to understand information about any health questions or concerns?	•Usually
Q18	seem to know about your medical history?	•Always
Q19	show respect for what you had to say?	
Q20	spend enough time with you?	

Items from the Consumer Assessment of Healthcare Providers and Systems (CAHPS) Clinician and Group Survey [18]

To capture patients’ assessment of shared decision-making, we will use selected items from the CAHPS patient-centered medical home supplement [19] (see Table 5).

**Table 5:** Shared decision-making questions

In the last 12 months, did you and your provider talk about starting or stopping a prescription medication? If yes, complete the rest of the questions.		Response options
PCMH7	When you talked about starting or stopping a prescription medicine, how much did your provider talk about the reasons you might want to take a medicine?	Not at all, a little, some, a lot
PCMH8	When you talked about starting or stopping a prescription medicine, how much did your provider talk about the reasons you might NOT want to take the medicine?	Not at all, a little, some, a lot
PCMH9	When you talked about starting or stopping a prescription medicine, did your provider ask you what you thought was best for you?	Yes, no

Items from Consumer Assessment of Healthcare Providers and Systems (CAHPS) patient-centered medical home supplement [19]

In addition, we will assess patients’ healthcare utilization and mortality using claims data. We will measure hospitalizations for stroke, acute myocardial infarction, and angina identified via primary diagnoses and in-hospital mortality for these conditions. For patients covered by Medicare and Medicaid, we will also be able to assess non-hospital related mortality. We will also collect clinical data on patients’ blood pressure, cholesterol levels, smoking status, and CVD risk based on the ASCVD risk calculator developed by the American Cardiology Foundation and the American Heart Association (see Table 6). These clinical data will be extracted and summarized by the CCNC Informatics Center.

**Table 6:** Practice-level patient outcomes

Outcome	Measure
Communication	Percentage of patients who answered “always” to all 6 communication questions
Shared decision-making (SDM)	Percentage of patients who answered “a lot” and “yes” to all 3 SDM questions.
Hospitalizations	Percentage of patients hospitalized for stroke, MI, angina.
In-hospital mortality	Percentage of patients hospitalized for stroke, MI, angina who died in the hospital.
Blood pressure	Percentage of patients with BP <140/90, <140/90 if 18–59 years, <150/90 if 60–85 years.
Systolic blood pressure	Percentage of patients with a SBP <140; mean (SD), median (IQR) SBP
Diastolic blood Pressure	Percentage of patients with DBP < 90; mean (SD), median (IQR) DBP
Low-density lipoprotein (LDL) level	Percentage of patients with LDL levels in the following categories: <100 mg/dL; 100 to <160 mg/dL; 160 mg/dL or more
10-year CVD Risk^a^	Mean (SD), median (IQR) of individuals who had CVD risk calculated

^a^based on ACSVD

We will measure practice outcomes qualitatively by asking whether evidence-based CVD prevention improved the practice’s standing in a pay-for-performance program, provided them with maintenance of certification credits, improved their payments through the BCBS of NC sponsored Blue Quality Physicians Program, or made them more attractive for recruitment into ACOs/Shared Savings Programs and other global payment programs.

*Inner context* refers to structural, political, and cultural features of the practice that are directly related to experiences of patients and staff, as well as features of the larger organization (if any) with which a practice is associated [20]. We will capture inner context through our measures of practice capacity for QI, organizational readiness, and implementation climate. In addition, we will assess other structural features, such as practice type (see Table 7).

**Table 7:** Inner context measures

Practice type	Solo, single-specialty, multispecialty (NAMCS-EMR Q11-13)
Degree of integration	Practice owned by physician/group, HMO, CHC, AMC, other hospital, other corp. (NAMCS-EMR Q22)
Practice size	Number of physicians (NAMCS-EMR Q12)
Practice staffing	Number of mid-level providers (NAMCS-EMR Q14)
Patient volume	Number of office visits in a normal week (NAMCS-EMR Q10)/per provider full time equivalent (FTE)
Payer mix	Percent patient care revenue from Medicare, Medicaid, private insurance, other (NAMCS-EMR Q23)
EMR capabilities	Number and use of computerized capabilities (NAMCS-EMR Q18a-Q18j)

Items from the 2010 National Ambulatory Medical Care Survey electronic medical records

*Outer context* refers to features of the economic, political, and social context within which an organization resides [20]. Through the KI survey, we will assess the perceived influence of external events using a 3-point Likert scale (no influence to significant influence). In KI interviews, we will explore qualitatively how events rated “2” (some influence) influenced the practice’s efforts to implement evidence-based CVD prevention processes of care.

### Data analysis

#### Aim 1: Evaluate the effect of primary care practice support on delivery of evidence-based CVD prevention

Our primary hypotheses are that primary care practice support will improve the percentage of patients in a practice: (a) with ischemic vascular disease who use aspirin or other antithrombotics; (b) with hypertension who have adequate blood pressure control; (c) with an LDL test performed who are prescribed a recommended dose of statin based on risk status; (d) who were screened for tobacco use and, if identified as a tobacco user, received cessation counseling and/or pharmacological therapy; and (e) who receive aspirin for primary prevention based on a 10-year CVD risk status. Our secondary hypotheses are as follows: (a) practice capacity for QI, organizational readiness to implement change, implementation climate, and contextual factors (inner and outer) will moderate the effect of primary care practice support on evidence-based CVD prevention; and (b) primary care practice support will reduce race-based disparities in evidence-based CVD prevention.

#### Statistical analysis

The statistical analysis of the primary outcomes for Aim 1 will be based on a generalized linear mixed model (GLMM) for longitudinal binomial data at the practice level. Allowing for the number of patients who are observed within a practice to vary across time, the models will be estimated by maximizing the pseudo-likelihood, specifying practices as clusters, and using empirical variance estimators. For example, the number of hypertensive patients whose blood pressure is under control *Y*_it_, out of *N*_it_ hypertensive patients, will follow a binomial (*N*_it_, *π*_it_) distribution where, conditional upon a practice-specific random intercept *b*_i0_ and slope *b*_i1_, π_it_ = *E*(*Y*_it_/*N*_it_|*b*_i0_,*b*_i1_) is the expected proportion of hypertensive patients whose blood pressure is controlled within the *i*-th practice in the *t*-th month; *t* = s_i_ (baseline), s_i_ + 3, s_i_ + 6, s_i_ +9, s_i_ + 12, s_i_ + 18 (6 month post-intervention follow-up), and for some practices s_i_ + 24 (12 month post-intervention follow-up). The GLMM is

1 $$ \mathrm{Logit}\left({\uppi}_{\mathrm{i}\mathrm{t}}\right) = {\beta}_0 + {b}_{\mathrm{i}0} + {\beta}_1{X}_{\mathrm{i}1} + {\beta}_2{X}_{\mathrm{i}2} + {\beta}_{12}{X}_{\mathrm{i}1}{X}_{\mathrm{i}2} + {g}_1\left({t}^{*};\uppsi \right) + {b}_{\mathrm{i}1}t + {g}_2\left(t;\uplambda \right){X}_{\mathrm{i}\mathrm{t}3} + {g}_3\left(t;\uptheta \right){X}_{\mathrm{i}\mathrm{t}4} $$

where *X*_i1_ = 1 indicates east region and 0 for west region; *X*_i2_ = 1 indicates higher readiness and 0 for lower readiness stratum; *g*_t_(t^*^;ψ) is a smooth spline function of calendar time (t^*^) for the secular trend corresponding to the control condition with finite dimensional parameter ψ; *X*_it3_ = 1 indicates the active (12 months) intervention period versus 0 otherwise and *g*_2_(*t*; λ) is function for four piecewise conjoined linear segments each of 3 months duration with λ being the vector of slope parameters; *X*_it4_ = 1 is an indicator for the maintenance period versus 0 otherwise and *g*_3_(*t*;θ) is a function for two piecewise conjoined linear segments the first of 6 months duration to which all practices will contribute data and the second 6 months durations to which only a subset of practices will contribute data as determined by the stepped-wedge design. Finally, *b*_i0_ and *b*_i1_ are assumed to be bivariately normal random variables with respective variances *σ*_0_^2^ and *σ*_1_^2^ and covariance *σ*_01_. Primary interest is in the treatment effect describing the log odds of a randomly sampled patient in a practice having the outcome 12 months post-intervention relative to the log odds of a randomly sampled patient from the same practice having the outcome during the pre-intervention period; this log odds ratio is well-defined as a function of λ parameters. Finally, the average causal effect of 12 months of intervention will be calculated as the average difference in proportions, Σ_i_[E(*Y*_it_/*N*_it_|*b*_i0_,*b*_i1_,*t* = s_i_ + 12)—E(*Y*_it_/*N*_it_|*b*_i0_,*b*_i1_*t* = s_i_)]/300; a 95 % confidence interval will be determined by bootstrapping. The random coefficients logistic model (1) will be fitted using SAS Proc GLIMMIX.

The secondary hypotheses will (a) introduce practice capacity for QI, organizational readiness to change, implementation climate, and contextual factors as main effects and effect modifiers in model (1) and introduce race as a main effect and effect modifier to examine whether primary care practice support reduces race-based disparities in the use of evidence-based CVD prevention. Examination of race will require outcome data aggregated at the practice level to be broken out by race.

#### Statistical power

Power to detect the effect of intervention after 12 months with respect to the outcome of hypertension control is simulated for the stepped-wedge study design assuming 300 practices, and an average of *μ* = 1200 or 1600 patients per practice (assuming a typical practice has 4 physicians with 300 or 400 patients per physician). For data collection months *t* = s_i_ (baseline), *s*_i_ + 3, *s*_i_ + 6, *s*_i_ +9, *s*_i_ + 12, and *s*_i_ + 18 (6 months post-intervention follow-up), power calculations are based on practice-month summaries consisting of 1800 binomial observations (6 × 300). Power is determined by simulating data from a model similar to model (1) but, for simplicity, assuming no temporal trend:

2 $$ \mathrm{logit}\left\{E\left({Y}_{\mathrm{i}\mathrm{t}}/{N}_{\mathrm{i}\mathrm{t}}\Big|{b}_{\mathrm{i}0},{b}_{\mathrm{i}1}\right)\right\} = {\beta}_0 + {b}_{\mathrm{i}0} + {\beta}_2{X}_{\mathrm{i}2} + {\beta}_3{X}_{\mathrm{i}\mathrm{t}3} + {b}_{\mathrm{i}1}t $$

where *X*_it3_ = 0 if *t* = s_i_; *X*_it3_ = (*t* – s_i_)/12 for *s*_i_ < *t* ≤ *s*_i_ + 12, and *X*_it3_ = 1 for *t* = *s*_i_ + 18. This model assumes a linear logistic trend during the active intervention period and a constant slope during the maintenance period equal to the 12-month intervention effect. Additionally, model (2) specifies a practice-specific intercept and slope assumed to be normally distributed with means 0 and variances *σ*_0_^2^ = 0.42, *σ*_1_^2^ = 0.32 and covariance *σ*_01_ = −0.15, values estimated from preliminary pretest-posttest data on 19 medical practices (not shown). Primary interest is in exp(*β*_3_), the practice-specific odds ratio of having a positive outcome (e.g., blood pressure controlled) after 12 months intervention relative to baseline. The number of patients in the i-th practice assumed to be constant across time (*N*_i_ = *N*_it_ for all *t*) was randomly selected from *N*_i_ ~ *N*(*μ*, *μ*^7/4^) to account for expected variability in practice size. A complete data set is then generated using model (2) with (*β*_0_ = 0.50, *β*_2_ = 0.05, *β*_3_ = 0.05) corresponding to a baseline of 62 % blood pressure control for least ready practices. The fraction of times that *H*_0_: *β*_3_ = 0 is rejected with two-sided *α* = 0.05 Wald tests under the correctly specified model was taken to be the simulated power for detecting a non-null odds ratio *β*_3_. When the true *β*_3_ = 0, the results represent simulated type I error, which is seen to be close to the nominal 0.05 significance level. The stepped-wedge design has excellent power for the outcome, percentage of hypertensive patients with controlled blood pressure (see Additional file 1).

#### Aim 2: Evaluate the effect of primary care practice support on patient-level health outcomes

Our primary hypotheses are that primary care practice support will improve the percentage of patients in a practice who indicate that their provider (a) always communicated well with them during the visit, and (b) involved them a lot of the times in shared decision-making about their medications. Our secondary hypotheses are that primary care practice support will decrease (a) the percentage of patients within a practice who were hospitalized for a cardiovascular disease-related condition, (b) the percentage of patients within a practice who were hospitalized for a cardiovascular disease-related condition or died, and (c) race-based disparities in CVD-related hospitalizations and mortality. We will explore whether primary care practice support improves the percentage of patients in a practice whose tobacco use status and overall cardiovascular risk were assessed, whose blood pressure (BP) was adequately controlled, and who received appropriate management with regard to aspirin use, tobacco cessation support, and use of statin therapy.

### Statistical analysis

For the primary hypotheses, communication and shared decision-making are binomial outcomes derived from patient surveys administered at baseline (*t* = s_i_) and at the end of the intervention period (*t* = s_i_ + 12). The model to assess improvement in these outcomes is a simplification of model (1) because there are only two repeated outcomes:

3 $$ \mathrm{Logit}\left({\uppi}_{\mathrm{i}\mathrm{t}}\right) = {\beta}_0 + {b}_{\mathrm{i}0} + {\beta}_1{X}_{\mathrm{i}1} + {\beta}_2{X}_{\mathrm{i}2} + {\beta}_{12}{X}_{\mathrm{i}1}{X}_{\mathrm{i}2} + {g}_1\left({t}^{*};\uppsi \right) + {b}_{\mathrm{i}1}t + {\beta}_3{X}_{\mathrm{i}\mathrm{t}3} $$

where *X*_it3_ = 1 if *t* = s_i_ + 12 (end-of-intervention period) and *X*_it3_ = 0 if *t* = s_i_ (baseline). Note that exp(*β*_3_) is the odds ratio of a positive response (e.g., always communicated well, involved them in shared decision-making a lot of the time) after 12 months of intervention relative to the odds of a positive response at baseline, controlling for secular trends via ψ. The average causal effect of 12 months of intervention versus the control condition will be calculated as the average difference in proportions as described for Aim 1.

For the secondary hypothesis, model (2) will apply to claims data for the percentage of patients (a) hospitalized for a cardiovascular disease-related condition and (b) hospitalized for a CVD-related condition or died. For these outcomes, *X*_it3_ = 1 if *t* = s_i_ + 18 (6 months post-intervention) and *X*_it3_ = 0 if *t* = s_i_ (baseline). To assess whether primary care practice support decreases race-based disparities in these outcomes, the binomial data will be obtained for each race and practice and time point. Other facility-level patient outcomes are given in Table 4. Model (3) will be fitted to all binomial outcomes (e. g., percentage of patients…) using SAS Proc GLIMMIX. For continuous outcomes (e.g., a 10-year CVD Risk), an identity link function leading to a linear model fitted with SAS PROC MIXED will be used with variance weight 1/*N*_it_ where *N*_it_ is the observed number of patients in the *i*-th practice at the *t*-th month.

#### Statistical power

Statistical power for the primary hypotheses is simulated using model (3) with *β*_1_ = *β*_12_ = 0 and dropping *g*_1_(*t*;ψ) from the model. It is based on a planned constant sample size (denominator) of *N* = 200 patients/practice at each time point. For the communication outcome, we assumed *β*_0_ = 1.4 corresponding to a baseline rate of 80 % for a low ready practice (*X*_i2_ = 0). For the shared decision-making outcome, we set *β*_0_ = −0.8 giving 30 % baseline when *X*_i2_ = 0. Finally, we fixed *β*_2_ = 0.05 and *β*_3_ = 0.05, variances *σ*_0_^2^ = 0.42 and *σ*_1_^2^ = 0.32, and covariance *σ*_01_ = −0.15. These values for variance components are adopted from Aim 1 for lack of preliminary data for Aim 2. They are considered reasonable estimates because the statistical analysis for Aim 2 involves a random effects logistic regression model for repeated binomial data. Power exceeds 98 % for detecting differences as small as 0.04 in proportions between intervention and control (see Additional file 2).

#### Aim 3: Examine the effect of primary care practice support on implementation of clinical practice and office system changes that support delivery evidence-based CVD prevention

Our primary hypothesis is that primary care practice support will increase implementation of clinical practice and office system changes to improve evidence-based CVD prevention. Our secondary hypotheses are that (a) primary care practice support intensity will increase implementation of clinical practice and office system changes, and (b) practice capacity for QI, organizational readiness to implement change, and contextual factors will moderate the effect of primary care practice support on the implementation of clinical practice and office system changes. We will explore the acceptability of primary care practice support from the perspective of the provider champion.

#### Statistical analysis

A random coefficients linear mixed model will be applied to the KDIS based on practice facilitator ratings collected monthly throughout the intervention period:

4 $$ E\left({Y}_{\mathrm{i}\mathrm{t}}\Big|\ {b}_{\mathrm{i}0},\ {b}_{\mathrm{i}1}\right) = {\beta}_0 + {b}_{\mathrm{i}0} + {\beta}_1{X}_{\mathrm{i}1} + {\beta}_2{X}_{\mathrm{i}2} + {\beta}_{12}{X}_{\mathrm{i}1}{X}_{\mathrm{i}2} + {g}_1\left({t}^{*};\uppsi \right) + {b}_{\mathrm{i}1}t + {g}_2\left(t;\uplambda \right)\ {X}_{\mathrm{i}\mathrm{t}3} $$

where the explanatory variables and parameters are defined as in model (1). In particular, *X*_it3_ = 1 for all *t* = s_i_ + 1,…,s_i_ + 12, and *g*_2_(*t*; λ) is function for four piecewise conjoined linear segments each of 3 months duration in the intervention period with λ being the vector of slope parameters, which we expect to be positive indicating an increase in the number of clinical practice and office system changes with an increase in the amount of implementation support. The main hypothesis is an overall positive change in expected outcome between 12 months post-intervention and 1 month post-intervention. The first secondary hypothesis is that primary care practice support intensity (with *X*_it3_ re-defined and scaled as a continuous measure ranging from 0 to 1) will be positively associated with the KDIS scale *Y*_it_. The second secondary hypothesis will assess practice capacity for QI, organizational readiness to implement change, and contextual factors as effect modifiers on the rates of implementation given by *g*_2_(*t*; λ).

#### Statistical power

Power is simulated using a mixed effects linear model similar to model (4) for the KDIS scale with *β*_1_ = *β*_12_ = 0 and dropping *g*_1_(*t*;ψ) giving an assumption for simplicity of no secular trend:

5 $$ E\left({Y}_{\mathrm{i}\mathrm{t}}\Big|\ {b}_{\mathrm{i}0},\ {b}_{\mathrm{i}1}\right) = {\beta}_0 + {b}_{\mathrm{i}0} + {\beta}_2{X}_{\mathrm{i}2} + {b}_{\mathrm{i}1}t + {\beta}_3{X}_{\mathrm{i}\mathrm{t}3} $$

with *X*_it3_ = (*t*-1)/3 for *t* = 1, 2, 3, and 4. Model (5) differs from model (4) in that we model 3-month averages of KDIS scales producing quarterly data, in accordance with preliminary data reported in Figure 2 of Halladay et al., which is as follows: electronic health record (mean 2.78, sd = 3.17); care protocols (mean 1.71, sd = 4.37); planned care (mean 2.16, sd = 3.84); and self-management support (mean = 1.38, 3.67). It follows their variances are 10, 19.1, 14.7, and 13.5, respectively [15]. Model (5) assumes that the intervention effect on KDIS is linear in time during the yearlong implementation period; under this model, the main hypothesis is an overall positive change in expected outcome between 12 months post-intervention and 3 months post-intervention (*β*_3_ > 0). For power calculations, we set *β*_0_ = 2, *β*_2_ = 0.5, *β*_3_ = 1.0, *σ*_0_^2^ = 5 (or 8), *σ*_1_^2^ = 4, *σ*_01_ = −2 and *σ*_e_^2^ = 5 (or 8). Then, under the random coefficients model, Var(*Y*_it_) = *σ*_0_^2^ + *σ*_1_^2^t^2^ + 2 t *σ*_01_ + *σ*_e_^2^. Additional file 3 shows that power to show an effect of intervention on KDIS scales exceeds 85 % under the assumptions.

#### Supplemental analysis

We will use simple statistics to describe the frequency and distribution of positively and negatively coded comments about the acceptability of primary care practice support. We will examine whether acceptability varied by primary care practice support intensity, baseline capacity for QI, and inner and outer contextual features. We will use the qualitative data to explore the reasons why KIs found primary care practice support acceptable (or not) and recommendations they have for future efforts.

#### Aim 4: Evaluate the effect of practice facilitation on practice capacity to implement future evidence-based clinical guidelines

Our primary hypothesis is that primary care practice support will improve capacity to implement other evidence-based clinical guidelines in the future. Our secondary hypotheses are that (a) contextual factors will moderate the effect of primary care practice support on practice capacity to implement additional evidence-based clinical guidelines, and (b) primary care practice support will generate additional positive practice outcomes.

#### Statistical analysis

We will analyze adaptive reserve and change process capability with a linear mixed model applied to the measures collected at baseline (*t* = s_i_), at the end of the intervention period (*t* = s_i_ + 12), and, for the early enrolled practices, at 6 months post-intervention (*t* = s_i_ + 18). The model to assess improvement in these outcomes is a simplification of model (1) because there are only two repeated outcomes:

6 $$ E\left({Y}_{\mathrm{i}\mathrm{t}}\Big|\ {b}_{\mathrm{i}0,}{b}_{\mathrm{i}1}\right) = {\beta}_0 + {b}_{\mathrm{i}0} + {\beta}_1{X}_{\mathrm{i}1} + {\beta}_2{X}_{\mathrm{i}2} + {\beta}_{12}{X}_{\mathrm{i}1}{X}_{\mathrm{i}2} + {g}_1\left({t}^{*};\uppsi \right) + {b}_{\mathrm{i}1}t + {\beta}_3{X}_{\mathrm{i}\mathrm{t}3}+{\beta}_4{X}_{\mathrm{i}\mathrm{t}4} $$

where *X*_it3_ = 1 if *t* = s_i_ + 12 (end-of-intervention period) and *X*_it3_ = 0 otherwise; *X*_it4_ = 1 if *t* = s_i_ + 18 (6 months post-intervention) and baseline is the reference time period. Other assumptions are as in model (4). Primary interest is in *β*_3_, the difference in mean measure between baseline and end-of-intervention period.

In secondary analyses, contextual factors will be examined as additional main effects and as statistical interactions with *X*_it3_ in order to assess whether there are effect modifiers for the effect of primary care practice support on practice capacity to implement future evidence-based clinical guidelines. To test the hypothesis that primary care practice support generated additional positive practice outcomes, we will calculate 95 % confidence intervals for proportions of practices reporting positive outcomes, both overall and stratified by practice characteristics (inner context). We will also use logistic regression to ascertain whether additional positive outcomes varied as a function of primary care practice support intensity, KDIS scores, ABCS measures, and inner context variables.

### Statistical power

For the primary hypotheses, we conservatively calculate power by ignoring any information from individuals 6 months post-intervention and allow a 15 % dropout (*N* = 255). Additional file 4 shows that our minimal detectable differences are similar to mean change reported by Nutting et al. and Solberg et al., respectively, for adaptive reserve and CPCQ [10, 11].

#### Supplemental analysis

We will explore the reasons why some practices benefited more than others by identifying themes about actions taken, missed opportunities, influential inner and outer contextual factors, and other situational factors.

#### Trial status

Practices are being recruited. The high-readiness practices randomized to start in month 9 will receive the intervention beginning January 2016.

## Discussion

### Significance

Practice facilitation, when combined with other quality improvement interventions, is an effective strategy for supporting the implementation of evidence-based guidelines in primary care settings [21]. However, no previous effort has conducted a controlled trial of practice facilitation at the scale or level of intensity that we propose. Results will have significant implications for healthcare payers, government agencies, and other stakeholders seeking to use practice facilitation, alone or combined with other quality improvement interventions, to support national initiatives to scale up and spread evidence-based clinical guidelines.

### Limitations

Recruiting and retaining a large number of practices to engage in a yearlong, comprehensive quality improvement effort poses significant challenges. Potential recruitment barriers include fear of financial risk, reluctance to share data, acquisition by larger health systems, and competing priorities. Potential retention barriers include loss of a key clinician, resignation of a practice manager, and financial difficulties. We will attempt to mitigate these barriers by using practice facilitation to help practice achieve other goals (e.g., PCMH recognition) or enhance billing for services. Also, CCNC and AHEC will leverage their strong relationships with hospitals and health systems to boost recruitment and prevent attrition.

Our stepped-wedge study design affords some control of the effects of secular trends and other factors unrelated to our comprehensive quality improvement strategy. We will supplement this by documenting and including in our statistical analysis temporal events that could affect the implementation and outcomes of the strategy. However, the effects of outer context factors can be challenging to measure and model.

#### Impact

If successful, our comprehensive quality improvement strategy will have a positive impact on both practices and patients. Hundreds of small- to medium-sized primary care practices, many located in rural and medically underserved areas, will be better prepared for the transition to value-based payment, wherein an increasing proportion of reimbursement is tied to performance on quality of care measures. Likewise, discernible reductions in cardiovascular risk among the estimated 900,000 adult patients served by participating practices would likely prevent thousands of cardiovascular events within 10 years.

## Additional files

Additional file 1:
**P+ower to detect difference in blood pressure control between intervention and control condition (** ***β***_**3**_ **)with** **two-sided** ***α*** **= 5.0 % GLMM Wald significance test based on 300 practices and 200 simulations.**
Additional file 2:
**Power to detect difference in proportions (**∆**) of patients for the two primary** **patient-level** **health outcomes between the intervention after 12 months of implementation and the control condition,** **two-sided** ***α*** **= 0.05, GLMM Wald significance test based on 200 patients/practice per time point in each of 300 practices.**
Additional file 3:
**Power to detect difference in mean KDIS score of 1.0 (i.e.,** ***β***_**3**_ **=1) in a practice comparing the intervention after 12 months of implementation and the control condition with** **two-sided** ***α*** **= 0.05 based on** **two-sided** **F-tests** **from linear mixed model analysis assuming 300 practices and varying dropout rate and variance.**
Additional file 4:
**Minimum detectable change from baseline to end of intervention for practice capacity measures, 80 % power, varying assumption about** **intra-practice** **correlation (ρ) based on** **two-sided** ***α*** **= .05 paired** ***t*** **test (** ***N*** **=255).**

## References

[1] Heidenreich PA, Trogdon JG, Khavjou OA, Butler J, Dracup K, Ezekowitz MD (2011). Forecasting the future of cardiovascular disease in the United States: a policy statement from the American Heart Association. Circulation.

[2] Million Hearts: the initiative. http://millionhearts.hhs.gov/aboutmh/overview.html. Accessed September 29 2015.

[3] North Carolina quick facts from the US Census Bureau. http://quickfacts.census.gov/qfd/states/37000.html. Accessed June 20 2014.

[4] America’s health rankings: North Carolina cardiovascular deaths. 2012. http://www.americashealthrankings.org/NC/CVDDeaths/2012. Accessed June 20 2014.

[5] Teal R, Bergmire DM, Johnston M, Weiner BJ (2012). Implementing community-based provider participation in research: an empirical study. Implement Sci.

[6] Weiner BJ (2009). A theory of organizational readiness for change. Implement Sci.

[7] Weiner BJ, Amick H, Lee SY (2008). Conceptualization and measurement of organizational readiness for change: a review of the literature in health services research and other fields. Med Care Res Rev.

[8] Weiner BJ, Belden CM, Bergmire DM, Johnston M (2011). The meaning and measurement of implementation climate. Implement Sci.

[9] Weiner BJ, Lewis MA, Linnan LA (2009). Using organization theory to understand the determinants of effective implementation of worksite health promotion programs. Health Educ Res.

[10] Solberg LI, Asche SE, Margolis KL, Whitebird RR (2008). Measuring an organization's ability to manage change: the change process capability questionnaire and its use for improving depression care. Am J Med Qual.

[11] Nutting PA, Crabtree BF, Stewart EE, Miller WL, Palmer RF, Stange KC (2010). Effect of facilitation on practice outcomes in the National Demonstration Project model of the patient-centered medical home. Ann Fam Med.

[12] Jaen CR, Ferrer RL, Miller WL, Palmer RF, Wood R, Davila M (2010). Patient outcomes at 26 months in the patient-centered medical home National Demonstration Project. Ann Fam Med.

[13] Shea CM, Jacobs SR, Esserman DA, Bruce K, Weiner BJ (2014). Organizational readiness for implementing change: a psychometric assessment of a new measure. Implement Sci.

[14] Klein KJ, Sorra JS (1996). The challenge of innovation implementation. Acad Manage Rev.

[15] Halladay JR, DeWalt DA, Wise A, Qaqish B, Reiter K, Lee SY (2014). More extensive implementation of the chronic care model is associated with better lipid control in diabetes. J Am Board Fam Med.

[16] Klein KJ, Sorra JS (1996). The challenge of implementation. Acad Manag Rev.

[17] Jacobs SR, Weiner BJ, Bunger AC (2014). Context matters: measuring implementation climate among individuals and groups. Implement Sci.

[18] Agency for Healthcare Research and Quality. Development of the Clinician & Group Surveys. https://cahps.ahrq.gov/surveys-guidance/cg/about/Develop-CG-Surveys.html. Accessed June 2 2014.

[19] Agency for Healthcare Research and Quality. Patient Experience Measures from the CAHPS® Clinician & Group Surveys. Document No. 1309. 2012. https://cahps.ahrq.gov/surveys-guidance/cg/cgkit/1309_CG_Measures.pdf. Accessed May 21 2014.

[20] Tomoaia-Cotisel A, Scammon DL, Waitzman NJ, Cronholm PF, Halladay JR, Driscoll DL (2013). Context matters: the experience of 14 research teams in systematically reporting contextual factors important for practice change. Ann Fam Med.

[21] Baskerville NB, Liddy C, Hogg W (2012). Systematic review and meta-analysis of practice facilitation within primary care settings. Ann Fam Med.

[22] Klein KJ, Dansereau F, Hall RJ (1994). Levels issues in theory development, data collection, and analysis. Acad Manag Rev.

[23] Klein KJ, Kozlowski SWJ (2000). From micro to meso: critical steps in conceptualizing and conducting multilevel research. Organ Res Methods.

[24] Klein KJ, Kozlowski SWJ (2000). From micro to meso: critical steps in conceptualizing and conducting multilevel research.

[25] LeBreton JM, James LR, Lindell MK (2005). Recent issues regarding r(WG), r*(WG), r(WG)(J), and r*(WG)(J). Organ Res Methods.

[26] LeBreton JM, Senter JL (2008). Answers to 20 questions about interrater reliability and interrater agreement. Organ Res Methods.

[27] Centers for Medicare and Medicaid Services. Physician Quality Reporting System (PQRS) Measure Specifications Manual for Claims and Registry Reporting of Individual Measures. 2013. http://www.facs.org/ahp/pqrs/2014/registry-indiv-measure-spec.pdf. Accessed May 21 2014.

