# Supplementary material for: Advancing heart health in North Carolina primary care: the Heart Health NOW study protocol
Source: Implement Sci. 2015 Nov 14;10:160. doi: 10.1186/s13012-015-0348-4 (PMC4650518; doi:10.1186/s13012-015-0348-4)
Supplement: Additional file 1: — P+ower to detect difference in blood pressure control between intervention and control condition ( β 3 )with two-sided α = 5.0 % GLMM Wald significance test based on 300 practices and 200 simulations. [file 13012_2015_348_MOESM1_ESM.docx]

| **Additional File 1. Power to detect difference in blood pressure control between intervention and control condition (β_3_) with two-sided α=5.0% GLMM Wald significance test based on 300 practices and 200 simulations^1^** | | | | | |
| --- | --- | --- | --- | --- | --- |
| Mean Number of hypertensive patients/practice^2^ | Amount of practice dropout^3^ | OR=1  (β_3_=1, ∆ = 0)^4^  Type I error | OR=1.035 (β_3_=0.035)  (∆ = 0.009) | OR=1.05 (β_3_=0.050)  (∆ = 0.012) | OR>1.1 (β_3_>0.095)  (∆ > 0.023) |
| 1200 | None | 0.046 | 0.83 | 0.98 | 1.00 |
| 1200 | 15% | 0.042 | 0.82 | 0.98 | 1.00 |
| 1200 | 30% | 0.048 | 0.80 | 0.97 | 1.00 |
| 1600 | None | 0.050 | 0.92 | 0.99 | 1.00 |
| 1600 | 15% | 0.053 | 0.93 | 0.99 | 1.00 |
| 1600 | 30% | 0.053 | 0.90 | 0.99 | 1.00 |

^1^Type I error is based on 1000 simulations. ^2^The number of patients in the i-th practice is generated from N(1200,495^2^) or N(1600,636^2^). ^3^ This is the cumulative percent of dropout, or the percent of practices that have an incomplete data with dropout rate assumed constant over the study period. ^4^∆ = difference in proportions between control (baseline) condition and intervention condition after 12 months of implementation for a typical practice.
