# Supplementary material for: Advancing heart health in North Carolina primary care: the Heart Health NOW study protocol
Source: Implement Sci. 2015 Nov 14;10:160. doi: 10.1186/s13012-015-0348-4 (PMC4650518; doi:10.1186/s13012-015-0348-4)
Supplement: Additional file 2: — Power to detect difference in proportions (∆) of patients for the two primary patient-level health outcomes between the intervention after 12 months of implementation and the control condition, two-sided α = 0.05, GLMM Wald significance test based on 200 patients/practice per time point in each of 300 practices. [file 13012_2015_348_MOESM2_ESM.docx]

| **Additional File 2. Power to detect difference in proportions (∆) of patients for the two primary patient-level health outcomes between the intervention after 12 months of implementation and the control condition, two-sided α=0.05, GLMM Wald significance test based on 200 patients/practice per time-point in each of 300 practices^1^** | | | | |
| --- | --- | --- | --- | --- |
| **Amount of practice dropout^2^** | **OR=1 (β_3_=0, ∆ = 0)**  **Type I error** | **OR=1.2 (β_3_=0.18)** | **OR=1.25 (β_3_=0.22)** | **OR>1.3 (β_3_>0.26)** |
| ***Outcome: provider always communicated well during visit (β_0_=1.4 giving 80% baseline for low ready practice)*** | | | | |
|  | (∆ = 0) | (∆ = 0.03) | (∆ = 0.035) | (∆ > 0.04) |
| None | 0.103 | 0.91 | 0.99 | 1.00 |
| 15% | 0.100 | 0.91 | 0.98 | 0.99 |
| 30% | 0.101 | 0.84 | 0.96 | 0.99 |
| ***Outcome: provider involved them a lot of the time in shared decision making (β_0_=-0.8 giving 30% baseline)*** | | | | |
|  | (∆ = 0) | (∆ = 0.04) | (∆ = 0.05) | (∆ > 0.06) |
| None | 0.096 | 0.99 | 1.00 | 1.00 |
| 15% | 0.081 | 0.99 | 1.00 | 1.00 |
| 30% | 0.071 | 0.98 | 1.00 | 1.00 |

^1^Type I error is based on 1000 simulations; simulation results show inflation of Type I error relative to the nomial 0.05 level, which indicates need for refinement of the estimation method for Aim 2. ^2^This is the percent of practices that dropped out prior to the end of the intervention period.
