# Supplementary material for: Advancing heart health in North Carolina primary care: the Heart Health NOW study protocol
Source: Implement Sci. 2015 Nov 14;10:160. doi: 10.1186/s13012-015-0348-4 (PMC4650518; doi:10.1186/s13012-015-0348-4)
Supplement: Additional file 3: — Power to detect difference in mean KDIS score of 1.0 (i.e., β 3 =1) in a practice comparing the intervention after 12 months of implementation and the control condition with two-sided α = 0.05 based on two-sided F-tests from linear mixed model analysis assuming 300 practices and varying dropout rate and variance. [file 13012_2015_348_MOESM3_ESM.docx]

| **Additional File 3. Power to detect difference in mean KDIS score of 1.0 (i.e., β_3_=1) in a practice comparing the intervention after 12 months of implementation and the control condition with two-sided α=0.05 based on two-sided F-tests from linear mixed model analysis assuming 300 practices and varying dropout rate and variance^1^** | | | |
| --- | --- | --- | --- |
| Dropout^2^ | Type I error^3^ | Variance=10;(σ_0_^2^ = σ_e_^2^ =5) | Variance=16;(σ_0_^2^ = σ_e_^2^ =8) |
| 0% | .048 | .96 | .87 |
| 15% | .048 | .96 | .87 |
| 30% | .055 | .94 | .85 |

^1^The variance of the outcome at 12 months of intervention is σ_0_^2^+σ_e_^2^.  ^2^This is the cumulative percent of dropout, or the percent of practices that have incomplete data with dropout rate per quarter assumed constant over the intervention period.  ^3^Type I error bases on assuming variance=10.
