# Supplementary material for: Advancing heart health in North Carolina primary care: the Heart Health NOW study protocol
Source: Implement Sci. 2015 Nov 14;10:160. doi: 10.1186/s13012-015-0348-4 (PMC4650518; doi:10.1186/s13012-015-0348-4)
Supplement: Additional file 4: — Minimum detectable change from baseline to end of intervention for practice capacity measures, 80 % power, varying assumption about intra-practice correlation (ρ) based on two-sided α = .05 paired t test ( N =255). [file 13012_2015_348_MOESM4_ESM.docx]

| **Additional File 4. Minimum detectable change from baseline to end of intervention for practice capacity measures, 80% power, varying assumption about intra-practice correlation (ρ) based on two-sided α=.05 paired t-test (N=255)** | | | | |
| --- | --- | --- | --- | --- |
|  |  | Minimum detectable difference | | |
| Measure | Published difference^1^ | ρ=0.3 | ρ=0.5 | ρ=0.7 |
| Adaptive reserve | 0.05 | 0.07 | 0.06 | 0.05 |
| CPCQ | 0.11 | 0.12 | 0.11 | 0.08 |

^1^Nutting et al. reported mean (sd) as 0.69 (0.35) and 0.74 (0.35), respectively, for baseline and post-intervention period for adaptive reserve; Solberg et al. reported mean (sd) as 0.85 (0.59) and 0.96 (0.60), respectively, for baseline and post-intervention period for “organizational factors, overall” of the CPCQ.
